# Impaired central sensitivity to thyroid hormone is associated with hypertriglyceridemia in euthyroid population

**DOI:** 10.3389/fendo.2025.1616907

**Published:** 2025-07-14

**Authors:** Yu Gong, Guojuan Wang, Qianqian Zhang, Xu Jiang, Zhangxiang Zhu, Ying Li

**Affiliations:** ^1^ Department of Endocrinology and Metabolism, the Third Affiliated Hospital of Anhui Medical University (The First People’s Hospital of Hefei), Hefei, Anhui, China; ^2^ Graduate School, Bengbu Medical University, Bengbu, Anhui, China

**Keywords:** thyroid hormone sensitivity, hypertriglyceridemia, thyroid feedback quantile-based index, thyroid-stimulating hormone index, thyrotropin thyroxine resistance index

## Abstract

**Background:**

We aimed to investigate the correlation between central thyroid hormone sensitivity and Hypertriglyceridemia (HTG) in euthyroid population.

**Methods:**

A total of 833 individuals who underwent physical examinations were randomly selected. Biochemical parameters including thyroid hormones (THs), liver and kidney functions, blood glucose, and blood lipids were measured. Central TH sensitivity was evaluated using the thyroid feedback quantile-based index (TFQI), thyroid-stimulating hormone index (TSHI) and thyrotropin thyroxine resistance index (TT4RI). ​​ We analyzed the relationship between central TH sensitivity and triglyceride (TG) level using smooth curve fitting and threshold effect analysis and trend tests in multiple regression equations.​

**Results:**

TSHI (β=0.158, *P*=0.0443) was positively correlated with TG, while TT4RI (β=0.018, *P*=0.0112, inflection point: 25.809) and TFQI (β=0.798, *P*=0.0066, inflection point: -0.194) were both positively correlated with TG before the inflection points. Subgroup analyses revealed these relationships were particularly pronounced in females (TT4RI β=0.026, *P*=0.0205, inflection point: 22.487; TFQI β=0.780, *P*=0.0133, inflection point: -0.142), males (TFQI β=1.954, *P*=0.0100, inflection point: -0.395), individuals with age <65 years (TT4RI β=0.019, *P*=0.0119, inflection point: 25.809; TFQI β=0.878, *P*=0.0060, inflection point: -0.206), and individuals with BMI<28kg/m² (TT4RI β=0.026, *P*=0.0090, inflection point: 21.515; TFQI β=0.735, *P*=0.0132, inflection point: -0.173), all showing positive correlations before the point correlations. Tests for trend in multiple regression equations showed that with the increased quartiles of TT4RI (OR=1.321, *P*=0.00118) and TSHI (OR=1.253, *P*=0.00784), the risk of HTG increased correspondingly. For per SD increase in TT4RI, the odds of HTG increased by 36.5% (OR=1.365, *P*=0.00703). For per SD increase in TSHI, the odds of HTG increased by 19.1% (OR=1.191, *P*=0.06648).

**Conclusion:**

Impaired central thyroid hormone sensitivity is associated with increased triglyceride in euthyroid population, this association is more pronounced in individuals with aged<65 years and BMI<28kg/m^2^. Impaired central thyroid hormone sensitivity may be an independent risk factor for hypertriglyceridemia.

## Introduction

Dyslipidemia is a chronic disease with significant public health implications. According to WHO estimates, the global prevalence of elevated total cholesterol among adults aged ≥25 years was 39% in 2008 (1). The overall pooled prevalence of dyslipidemia in Chinese adults has reached 41.9% (2). Most dyslipidemia guidelines focused on low-density lipoprotein cholesterol (LDL-C), whereas triglycerides (TGs) have received comparatively less attention. With an estimated prevalence of 10% (3), Hypertriglyceridemia (HTG) significantly elevated the risk of atherosclerotic cardiovascular disease (ASCVD) through triglyceride-rich lipoproteins (TRLs) and their remnants (4). Furthermore, severe HTG independently increased pancreatitis risk (4).

Despite these clinical consequences, significant gaps persisted in identifying specific risk factors and high-risk individuals for HTG. Current risk assessment strategies failed to address ​​the distinct molecular pathophysiology of HTG. It has been established that endocrine hormones, such as thyroid hormones (THs), have emerged as key modulators of lipid metabolism (5).

THs critically regulate the synthesis, mobilization, and degradation of lipid ​​primarily via thyroid receptor β (TRβ), modulating sterol regulatory element-binding proteins (SREBPs) and peroxisome proliferator-activated receptor gamma coactivator 1-alpha (PGC-1α) pathways (6). A large body of evidence has revealed that fluctuations in either high or low levels of thyroid hormones could lead to dyslipidemia. In hypothyroidism, the levels of total cholesterol (TC), low-density lipoprotein cholesterol (LDL-C) and TG were increased, while the levels of high-density lipoprotein cholesterol (HDL-C) were decreased. In contrast, opposite changes in lipid profile were found in hyperthyroidism (7, 8). When thyroid stimulating hormone (TSH) was in the normal range, THs were positively correlated with the levels of TC, TG and LDL-C, but negatively correlated with the levels of HDL (9, 10). These findings highlighted the important role of THs on lipid regulation. Considering the complexity of hormone interactions between hypothalamic-pituitary-thyroid axis (HPA) and the specific metabolic behavior of thyroid hormones, thyroid function could not be comprehensively evaluated by a single serological index. However, thyroid hormone sensitivity index provided a new dimension for clinical research. Spanish researcher Laclaustra et al. proposed the thyroid feedback quantile-based index (TFQI), which reflected the sensitivity of central nervous system to THs (11). It was used to evaluate the response of pituitary to THs. The central thyroid hormone sensitivity indexes also included thyrotropin thyroxine resistance index (TT4RI) and thyroid-stimulating hormone index (TSHI) (12). This positions central TH sensitivity as a potential modulator of TG metabolism beyond classical pathways—a gap not addressed in prior epidemiological studies.

To our knowledge, one prior study has preliminarily explored the association between TFQI and TG in patients with nonalcoholic fatty liver disease (NAFLD) (13), but no research has comprehensively examined the relationship between central TH sensitivity and HTG risk in general euthyroid population. The purpose of our study was to investigate the association between central thyroid hormone sensitivity and HTG in euthyroid population, which may provide new ideas for early prevention for HTG in our clinic work.

## Materials and methods

### Participants

**Figure 1:**
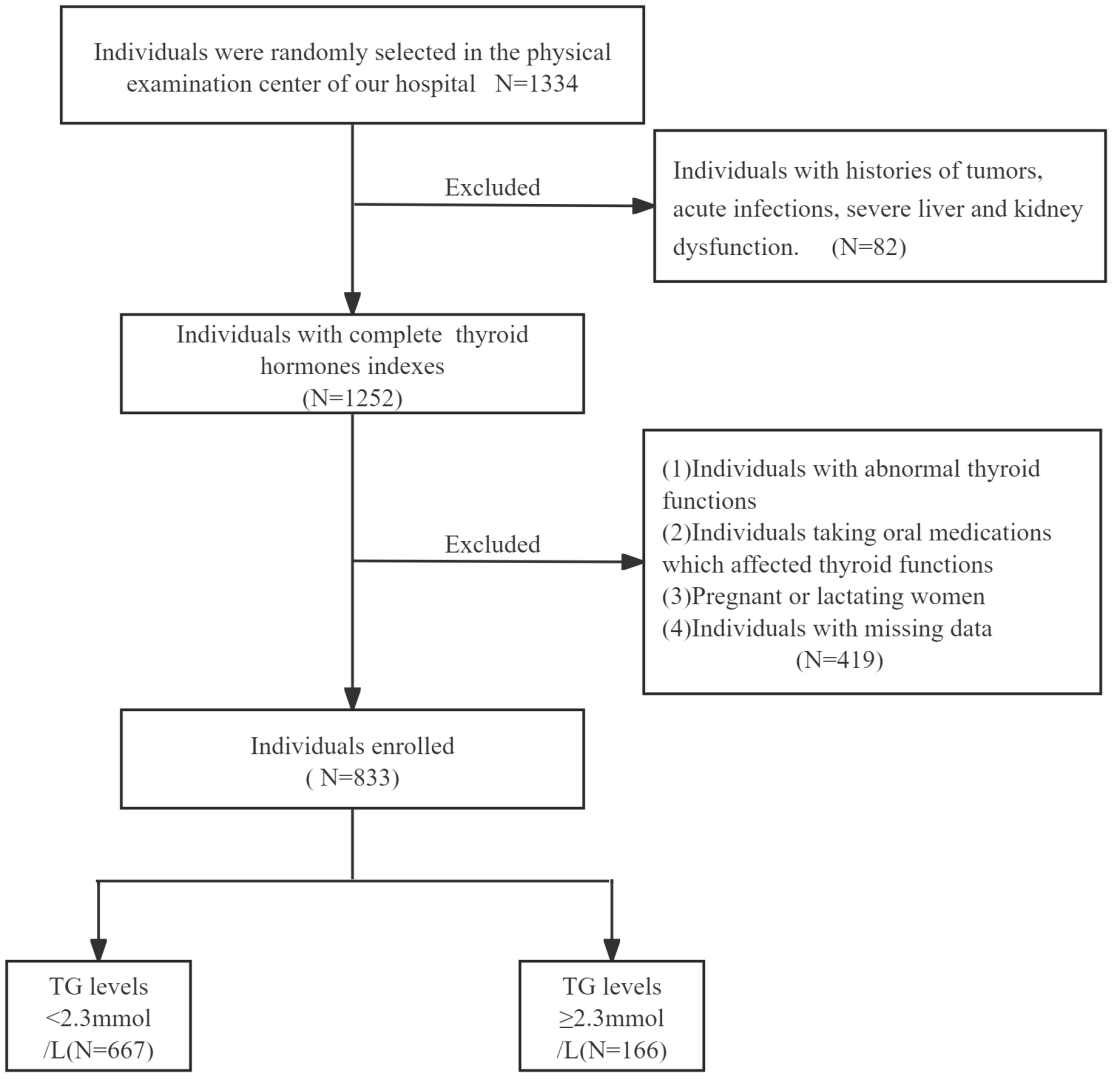
The flow chart of the study population.

We selected 1334 participants who underwent physical examinations in the physical examination center of the First People’s Hospital of Hefei from January 2024 to June 2024. According to the exclusion criteria and inclusion criteria, 833 individuals with normal thyroid functions were finally screened (**Figure 1**). There were 166 cases (115 males and 51 females, mean age: 46.36 ± 10.45 years) in TG≥2.3mmol/L group and 667 cases (331 males and 336 females, mean age: 47.66 ± 12.29 years) in TG<2.3mmol/L group.

Inclusion criteria: age between 18 and 90 years old (including 18 and 90 years old).

Exclusion criteria: (1) Individuals with histories of tumors, acute infections, severe liver and kidney dysfunction. (2) Individuals with abnormal thyroid functions, histories of thyroid diseases, including hyperthyroidism, hypothyroidism and hashimoto’s thyroiditis. (3) Individuals taking oral medications which affected thyroid functions, such as corticosteroids or thyroid hormones. (4) Pregnant or lactating women. (5) Individuals with missing data.

### Physical examination and laboratory measurements

The basic clinical information of all the individuals was collected: name, gender, age, chronic medical histories of diabetes mellitus (DM), hypertension, hyperlipidemia, drug use and surgery. Height, weight, systolic blood pressure (SBP) and diastolic blood pressure (DBP) were measured in the state of fasting in the morning. The levels of fasting blood-glucose (FBG), TG, TC, HDL-C, LDL-C, alanine transaminase (ALT), aspartic transaminase (AST), gamma-glutamyl transferase (GGT), total bilirubin (TBIL), direct bilirubin (DBIL), indirect bilirubin (IBIL), albumin (ALB), serum creatinine (SCr), serum uric acid (SUA), estimated glomerular filtration rate(eGFR), hemoglobin (Hb), blood platelets (Plt) were all measured by well-trained technicians (Roche automatic biochemical analyzer. Combas 8000). Thyrotropic hormone (TSH), free triiodothyronine (FT3) and free thyroxine (FT4) were determined using standardized Chemiluminescence methods (Abbott 12000SR). The normal ranges of THs: TSH (0.350-4.949μIU/mL) and FT4 (9.03-19.04pmol/L) and FT3 (2.43-6.01pmol/L). All measurements were performed using the same assays.

Hypertension was defined as SBP≥140mmHg or DBP≥90mmHg or self-reported histories (14). Diabetes was defined as FBG≥7.0mmol/L or self-reported history (15). HTG was defined as ≥2.3mmol/L (16).

Central TH sensitivity indices were calculated as follows: TT4RI = FT4 (pmol/L) × TSH (mIU/L) (17); TSHI = lnTSH (mIU/L) + 0.1345 × FT4 (pmol/L) (18); TFQI = cdfFT4 − (1 − cdfTSH): The index varies within the range of -1 to 1, with a negative value suggesting increased central TH sensitivity and a positive value suggesting decreased central thyroid hormone sensitivity.

### Statistical analysis

Sample size was calculated using G*Power 3.1 (19) to detect a clinically significant difference in TT4RI between TG<2.3mmol/L group and TG≥2.3mmol/L groups. Based on preliminary data (TG<2.3mmol/L group mean = 22.99, TG≥2.3mmol/L group mean = 27.24, pooled SD = 12.37; Cohen’s d = 0.34) with α = 0.05 (two-tailed), 90% power, and 1:4 case-control ratio, 166 cases of TG≥2.3mmol/L group and 667 cases of TG<2.3mmol/L group were required. This design provided >99% power to detect associations (OR ≥1.3) while maintaining EPV >15 for robust adjustment of 10 covariates. We used IBM SPSS Statistics Software Version 27, GraphPad Prism 10.0.3, Empower(R) (www.empowerstats.com, X&Y Solutions, Inc.) (Boston, MA), and R (http://www.r-project.org) to complete the statistical analyses. A significance level of *P*<0.05 was considered statistically significant. The normality of the data was assessed using the Shapiro-Wilk test and Q-Q plot. Normally distributed variables were presented as mean ± standard deviation (SD), while skewed variables were expressed as median (25th percentile, 75th percentile). Independent sample T-test was used to analyze the differences of central TH sensitivity indexes between TG<2.3mmol/L group and TG≥2.3mmol/L group. Smoothing function and threshold effect analysis were performed to determine the correlations of TFQI, TT4RI, TSHI and TG. Log-likelihood ratio tests for single-line linear regression model and two-segment linear regression model were conducted. Multiple regression equations were further tested for *P-*trend. All tests were two-way tests, and the statistical significance level was α=0.05. *P*<0.05 was considered statistically significant.

## Results

### The baseline clinical characteristics of all the individuals in this study

**Figure 2:**
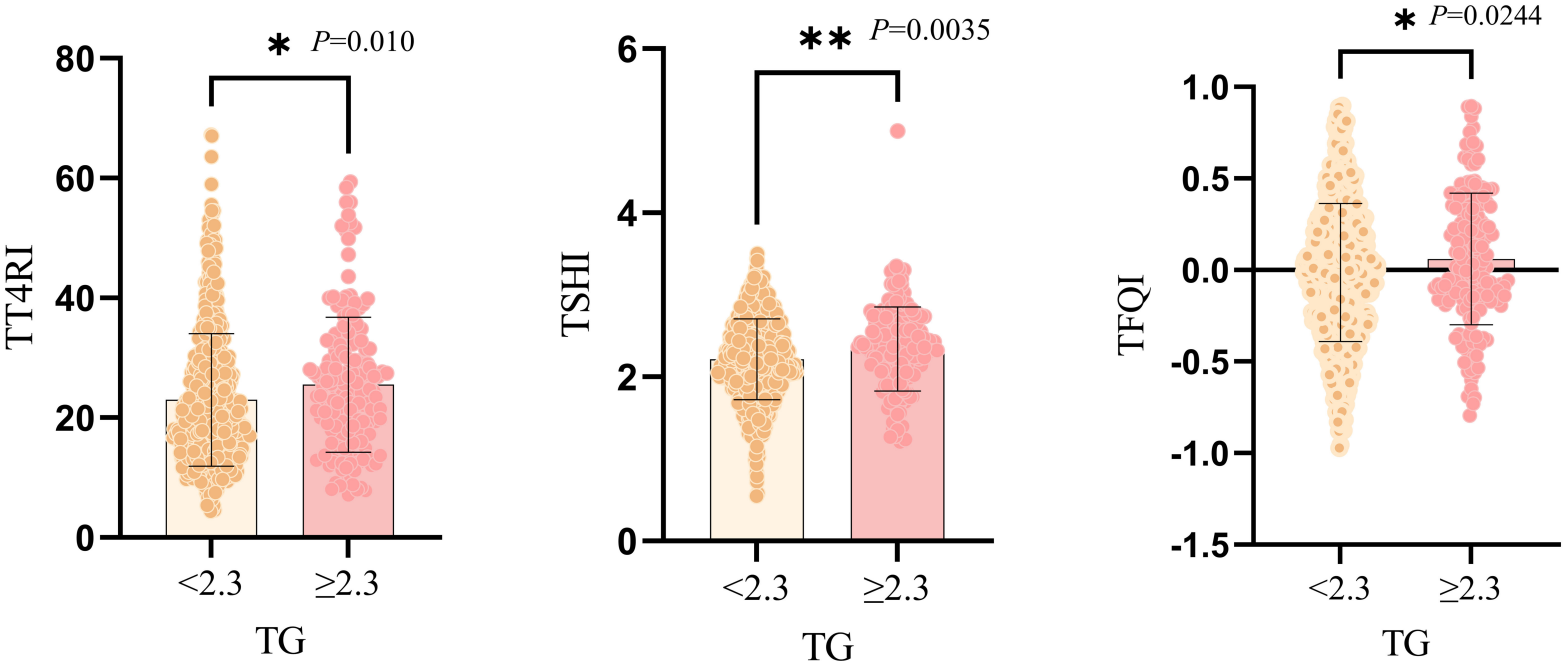
Graphical representation of independent samples T-test for comparison between two groups of TG subgroups. *:P<0.05; **:P<0.01.

Compared with TG<2.3mmol/L group, TG≥2.3mmol/L group showed higher proportion of women, younger ages and higher levels of BMI. The levels of FBG, ALB, ALT, AST, GGT, SUA, SCr and TC were significantly elevated. In terms of thyroid function, compared with TG<2.3mmol/L group, the levels of TSH, FT3, TT4RI, TSHI and TFQI were higher in TG≥2.3mmol/L group. There was no significant difference in FT4 level between two groups (*P*>0.05) (**Table 1**, **Figure 2**).

**Table 1:** Baseline characteristics of participants according to TG levels.

Characteristics	Total N=833	TG levels(mmol/L)		*P-*value
		<2.3 N=667	≥2.3 N=166	
Age (year)	47.40 ± 11.95	47.66 ± 12.29	46.36 ± 10.45	0.165
Male	446(53.5)	331(49.60)	115(69.3)	<0.001
Female	387(46.5)	336(50.40)	51(31.7)	<0.001
BMI (kg/m^2^)	24.83 ± 3.27	24.41 ± 3.20	26.55 ± 2.97	<0.001
SBP (mmHg)	122.98 ± 16.65	121.67 ± 16.68	128.22 ± 15.51	<0.001
DBP (mmHg)	75.35 ± 10.92	74.23 ± 10.81	79.86 ± 10.19	<0.001
FBG (mmol/L)	5.47 ± 1.40	5.39 ± 1.26	5.80 ± 1.85	0.007
ALB (g/L)	47.17 ± 2.82	47.06 ± 2.91	47.61 ± 2.38	0.024
ALT (U/L)	24.56 ± 18.10	22.34 ± 15.61	33.49 ± 23.83	<0.001
AST (U/L)	24.12 ± 8.16	23.28 ± 7.06	27.48 ± 10.97	<0.001
GGT (U/L)	34.94 ± 35.81	29.61 ± 29.96	56.36 ± 47.62	<0.001
TBIL (umol/L)	19.25 ± 7.58	19.63 ± 7.63	17.71 ± 7.21	0.003
IBIL (umol/L)	15.97 ± 6.40	16.21 ± 6.46	15.01 ± 6.11	0.031
DBIL (umol/L)	3.28 ± 1.43	3.43 ± 1.43	2.70 ± 1.27	<0.001
SCr (umol/L)	71.92 ± 16.14	70.87 ± 16.06	76.14 ± 15.81	<0.001
SUA (umol/L)	329.42 ± 92.32	317.36 ± 86.85	377.89 ± 97.87	<0.001
TG (mmol/L)	1.72 ± 1.21	1.27 ± 0.49	3.54 ± 1.49	<0.001
TC (mmol/L)	4.85 ± 0.90	4.78 ± 0.88	5.14 ± 0.92	<0.001
LDL-C (mmol/L)	3.06 ± 0.81	3.07 ± 0.80	3.03 ± 0.84	0.573
HDL (mmol/L)	1.33 ± 0.35	1.40 ± 0.34	1.04 ± 0.19	<0.001
Hb(g/L)	141.39 ± 17.08	139.54 ± 16.82	148.84 ± 16.13	<0.001
Plt (10*9/L)	224.43 ± 55.69	223.38 ± 54.87	228.67 ± 58.84	0.273
TSH (μIU/mL)	1.84 ± 1.09	1.78 ± 0.88	2.09 ± 1.67	0.022
FT3 (pmol/L)	4.43 ± 0.51	4.40 ± 0.50	4.55 ± 0.51	<0.001
FT4(pmol/L)	13.01 ± 1.45	13.02 ± 1.46	12.98 ± 1.44	0.789
TT4RI	23.81 ± 14.68	22.99 ± 11.06	27.10 ± 24.07	0.001
TSHI	2.24 ± 0.50	2.21 ± 0.49	2.34 ± 0.51	0.003
TFQI	0 ± 0.37	-0.01 ± 0.38	0.06 ± 0.36	0.024

Data are expressed as means ± standard deviations, medians (interquartile ranges), or percentages. Independent samples T-test or Mann-Whitney U-test are used to compare the differences of continuous variables between two groups.

### Smoothing function and threshold effect analysis of the relationship between central TH sensitivity and TG

**Figure 3:**
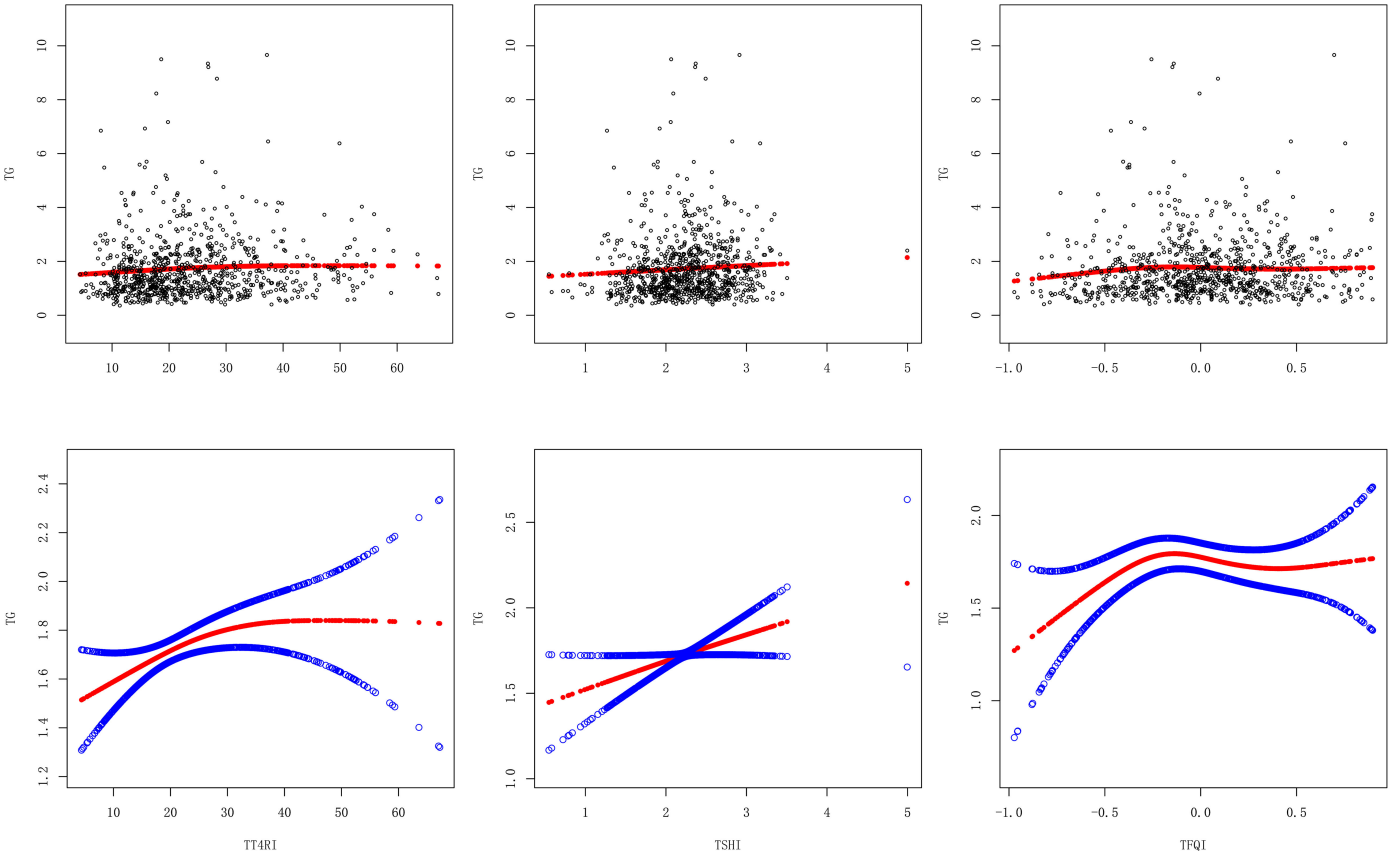
Smooth curve fitting diagram of central thyroid hormone sensitivity and TG.

After adjusting the confounding factors of gender, age, BMI, FBG, ALT, AST, GGT, TBIL, SUA and SCr, TT4RI was curvilinearly correlated with TG. TT4RI was positively correlated with TG before the inflection point at 6.471, with a corresponding increase of 0.018 in TG for every 1-unit increase in TT4RI (β=0.018, *P*=0.0112), there was no correlation after the inflection point. TSHI was positively correlated with TG, with a corresponding increase of 0.158 in TG for every 1-unit increase in TSHI (β=0.158, *P*=0.0443). TFQI was positively correlated with TG before the inflection point at -0.194, with a corresponding increase of 0.798 in TG for every 1-unit increase in TT4RI (β=0.798, *P*=0.0066), there was no correlation after the inflection point (*P*=0.1955) (**Table 2**, **Figure 3**).

**Table 2:** Threshold effect analysis of the relationship between central thyroid hormone sensitivity and TG in overall population.

Outcome: TG			
For exposure	TT4RI	TSHI	TFQI
Model I			
One-line effect	0.005 (-0.000, 0.010) 0.0715	0.158 (0.004, 0.312) 0.0443	0.100 (-0.106, 0.306) 0.3416
Model II			
Inflection point (k)	25.809	2.346	-0.194
< K segment effect1	0.018 (0.004, 0.032) 0.0112	0.324 (0.066, 0.582) 0.0139	0.798 (0.224, 1.373) 0.0066
> K segment effect2	0.000 (-0.007, 0.007) 0.9417	-0.080 (-0.415, 0.255) 0.6391	-0.206 (-0.519, 0.106) 0.1955
Difference in effect of 2 and 1	-0.018 (-0.035, -0.000) 0.0449	-0.404 (-0.909, 0.100) 0.1166	-1.005 (-1.777, -0.233) 0.0109
Log-likelihood ratio test	0.044	0.115	0.010

Outcome: β(95%CI) *P*-value, adjusted for gender, age, BMI, FBG, ALT, AST, GGT, TBIL, SUA and SCr.

### Subgroup analysis of the correlation between central thyroid hormone sensitivity and TG

In female group, after adjusting the confounding factors including age, BMI, FBG, ALT, AST, GGT, TBIL, SUA and SCr, TT4RI was positively correlated with TG before the inflection point at 22.487, with a corresponding increase of 0.026 in TG for every 1-unit increase in TT4RI (β=0.026, *P*=0.0205), there was no correlation after the inflection point. TFQI was positively correlated with TG before the inflection point at -0.142, with a corresponding increase of 0.780 in TG for every 1-unit increase in TFQI (β=0.780, *P*=0.0133), there was no correlation after the inflection point. TSHI had no correlation with TG. In male group, TFQI was positively correlated with TG before the inflection point at -0.395, with a corresponding increase of 1.954 in TG for every 1-unit increase in TT4RI (β=1.954, *P*=0.0100), there was no correlation after the inflection point. There were no correlations between TT4RI, TSHI and TG.

**Figure 4:**
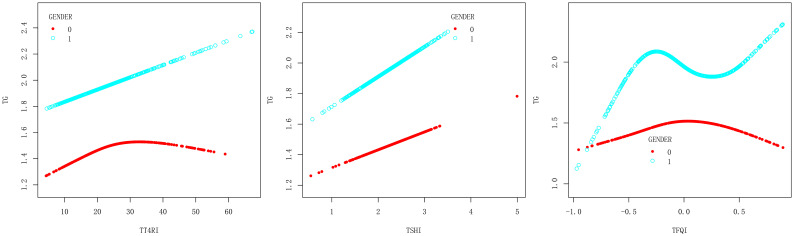
Smooth curve fitting diagram of TFQI, TT4RI, TSHI and TG were analyzed stratified by gender (0, female; 1, male).

We concluded that TT4RI and TFQI were positively correlated with TG in females, while TFQI was positively correlated with TG in males (**Table 3**, **Figure 4**).

**Table 3:** Gender subgroup analysis of the correlation between central thyroid hormone sensitivity and TG.

For exposure	TT4RI		TSHI		TFQI	
gender subgroup	female	male	female	male	female	male
Model I						
One-line effect	0.004 (-0.002, 0.009) 0.1859	0.011 (-0.000, 0.022) 0.0527	0.140 (-0.044, 0.324) 0.1356	0.227 (-0.014, 0.468) 0.0655	0.037 (-0.215, 0.289) 0.7745	0.189 (-0.125, 0.503) 0.2392
Model II						
Inflection point (k)	22.487	37.164	2.315	1.373	-0.142	-0.395
< K segment effect1	0.026 (0.004, 0.047) 0.0205	0.016 (0.001, 0.032) 0.0352	0.400 (0.068, 0.733) 0.0187	1.016 (-0.922, 2.953) 0.3049	0.780 (0.165, 1.395) 0.0133	1.954 (0.474, 3.434) 0.0100
> K segment effect2	0.000 (-0.006, 0.006) 0.9214	-0.006 (-0.040, 0.027) 0.7158	-0.157 (-0.523, 0.209) 0.4013	0.186 (-0.076, 0.447) 0.1644	-0.396 (-0.808, 0.016) 0.0605	-0.096 (-0.486, 0.294) 0.6302
Difference in effect of 2 and 1	-0.025 (-0.050, -0.001) 0.0392	-0.023 (-0.065, 0.020) 0.2983	-0.557 (-1.151, 0.037) 0.0667	-0.830 (-2.854, 1.194) 0.4219	-1.176 (-2.064, -0.287) 0.0099	-2.050 (-3.730, -0.369) 0.0172
Log-likelihood ratio test	0.037	0.294	0.064	0.417	0.009	0.016

Outcome: β(95%CI) *P*-value, adjusted for age, BMI, FBG, ALT, AST, GGT, TBIL, SUA and SCr.

In age<65 years group, after adjusting the confounding factors including gender, BMI, FBG, ALT, AST, GGT, TBIL, SUA and SCr, TT4RI was positively correlated with TG before the inflection point at 25.809, with a corresponding increase of 0.019 in TG for every 1-unit increase in TT4RI (β=0.019, *P*=0.0119), there was no correlation after the inflection point. TSHI was positively correlated with TG, with a corresponding increase of 0.177 in TG for every 1-unit increase in TSHI (β=0.177, *P*=0.0359). TFQI was positively correlated with TG before the inflection point at -0.206, with a corresponding increase of 0.878 in TG for every 1-unit increase in TFQI (β=0.878, *P*=0.0060), there was no correlation after the inflection point. In age≥65 years group, there were no correlations between TT4RI, TSHI, TFQI and TG.

**Figure 5:**
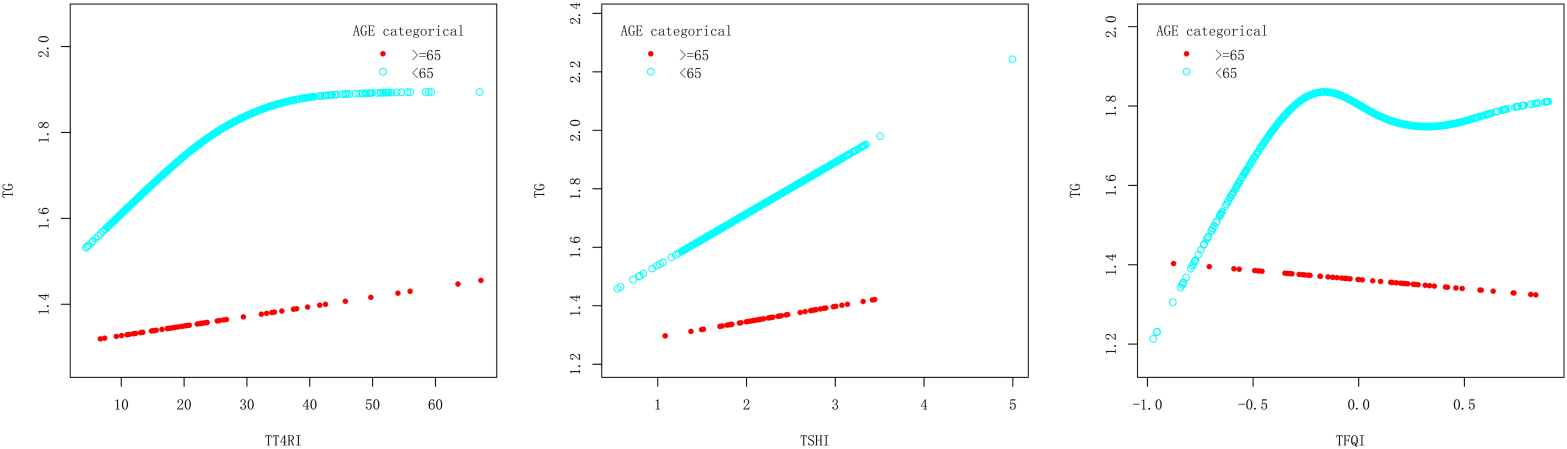
Smooth curve fitting diagram of TFQI, TT4RI, TSHI and TG were analyzed stratified by age.

We concluded that central TH sensitivity was positively associated with TG only in population with age<65 years (**Table 4**, **Figure 5**).

**Table 4:** Age subgroup analysis of the correlation between central thyroid hormone sensitivity and TG.

For exposure	TT4RI		TSHI		TFQI	
age subgroup	>=65	<65	>=65	<65	>=65	<65
Model I						
One-line effect	0.006 (-0.006, 0.018) 0.3460	0.005 (-0.000, 0.011) 0.0672	0.160 (-0.152, 0.472) 0.3185	0.177 (0.012, 0.341) 0.0359	0.086 (-0.332, 0.503) 0.6892	0.119 (-0.101, 0.339) 0.2883
Model II						
Inflection point (k)	23.587	25.809	2.247	2.346	0.636	-0.206
< K segment effect1	0.022 (-0.015, 0.058) 0.2475	0.019 (0.004, 0.033) 0.0119	0.352 (-0.268, 0.971) 0.2703	0.333 (0.061, 0.606) 0.0168	-0.141 (-0.608, 0.327) 0.5584	0.878 (0.254, 1.502) 0.0060
> K segment effect2	0.000 (-0.017, 0.017) 0.9690	0.001 (-0.007, 0.008) 0.8726	0.007 (-0.522, 0.537) 0.9785	-0.056 (-0.418, 0.307) 0.7630	4.572 (0.014, 9.130) 0.0541	-0.205 (-0.536, 0.127) 0.2270
Difference in effect of 2 and 1	-0.021 (-0.067, 0.025) 0.3696	-0.018 (-0.036, -0.000) 0.0489	-0.344 (-1.305, 0.616) 0.4851	-0.389 (-0.929, 0.151) 0.1584	4.713 (-0.056, 9.481) 0.0576	-1.083 (-1.917, -0.249) 0.0111
Log-likelihood ratio test	0.336	0.048	0.454	0.156	0.042	0.011

Outcome: β(95%CI) *P*-value, adjusted for gender, BMI, FBG, ALT, AST, GGT, TBIL, SUA and SCr.

In BMI<28kg/m^2^ group, after adjusting the confounding factors including gender, age, FBG, ALT, AST, GGT, TBIL, SUA and SCr, TT4RI was positively correlated with TG before the inflection point at 21.515, with a corresponding increase of 0.026 in TG for every 1-unit increase in TT4RI (β=0.026, *P*=0.0090), there was no correlation after the inflection point. TSHI was positively correlated with TG, for every 1-unit increase in TSHI, TG increased by 0.188 (β=0.188, *P*=0.0247). TFQI was positively correlated with TG before the inflection point at -0.173, with a corresponding increase of 0.735 in TG for every 1-unit increase in TFQI (β=0.735, *P*=0.0132), there was no correlation after the inflection point. In BMI≥28 kg/m^2^ group, there were no correlations between TT4RI, TSHI, TFQI and TG.

**Figure 6:**
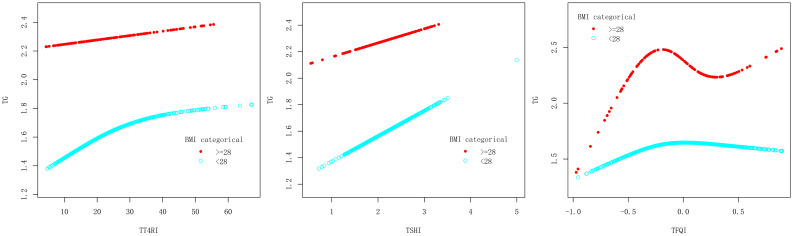
Smooth curve fitting diagram of TFQI, TT4RI, TSHI and TG were analyzed stratified by BMI.

We concluded that central TH sensitivity was positively associated with TG in population with BMI<28kg/m^2^ (**Table 5**, **Figure 6**).

**Table 5:** BMI subgroup analysis of the correlation between central thyroid hormone sensitivity and TG.

For exposure	TT4RI		TSHI		TFQI	
BMI subgroup	>=28	<28	>=28	<28	>=28	<28
Model I						
One-line effect	0.008 (-0.012, 0.028) 0.4546	0.005 (0.000, 0.011) 0.0440	0.190 (-0.248, 0.629) 0.3961	0.188 (0.024, 0.352) 0.0247	0.245 (-0.387, 0.877) 0.4488	0.090 (-0.125, 0.306) 0.4113
Model II						
Inflection point (k)	26.889	21.515	1.318	2.335	-0.257	-0.173
< K segment effect1	0.026 (-0.017, 0.070) 0.2414	0.026 (0.007, 0.046) 0.0090	0.983 (-1.540, 3.506) 0.4466	0.372 (0.091, 0.653) 0.0097	1.827 (-0.077, 3.730) 0.0622	0.735 (0.155, 1.314) 0.0132
> K segment effect2	-0.008 (-0.046, 0.030) 0.6813	0.002 (-0.005, 0.008) 0.6096	0.111 (-0.393, 0.616) 0.6660	-0.054 (-0.397, 0.289) 0.7576	-0.381 (-1.330, 0.567) 0.4322	-0.205 (-0.532, 0.122) 0.2202
Difference in effect of 2 and 1	-0.034 (-0.106, 0.037) 0.3501	-0.025 (-0.047, -0.002) 0.0310	-0.871 (-3.604, 1.861) 0.5331	-0.426 (-0.956, 0.104) 0.1157	-2.208 (-4.717, 0.301) 0.0868	-0.940 (-1.725, -0.155) 0.0193
Log-likelihood ratio test	0.335	0.03	0.52	0.113	0.077	0.019

Outcome: β(95%CI) *P*-value, adjusted for gender, age, FBG, ALT, AST, GGT, TBIL, SUA and SCr.

### Trend test of multiple regression equations for the relationship between central thyroid hormone sensitivity and HTG

**Figure 7:**
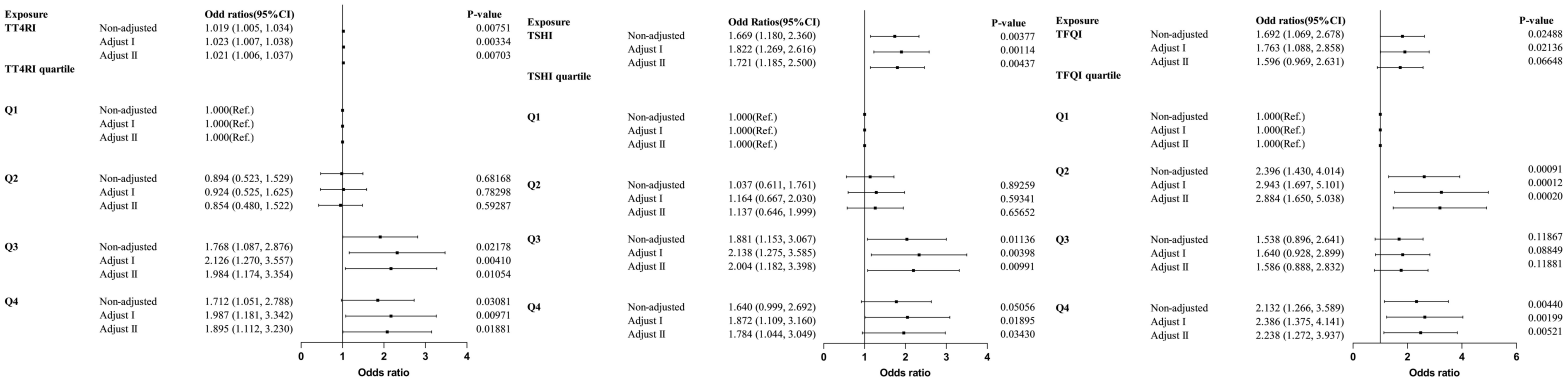
Forest plot of correlation between central thyroid hormone sensitivity and HTG.

Trend tests of multiple regression equations were performed to verify whether there was correlation between thyroid hormone sensitivity and HTG. HTG was defined as TG≥2.3mmol/L, Model I was adjusted for confounding factors including gender, age and BMI, Model II was adjusted for confounding factors including gender, age, BMI, FBG, ALT, AST, GGT, TBIL, SUA and SCr. The results showed that with the increase of quartiles of TT4RI and TSHI, the risk of HTG increased significantly. With per quartile increase in TT4RI, the hazard ratio for HTG increased by 32.1% (OR=1.321, *P*=0.00118). For per SD increase in TT4RI, the risk of HTG increased by 36.5% (OR=1.365, *P*=0.00703). The risk of HTG in the highest quartile of TT4RI was 1.895 times higher than that in the lowest quartile (OR=1.895, *P*=0.01881). With per quartile increase in TSHI, the hazard ratio for HTG increased by 25.3% (OR=1.253, *P*=0.00784). For per SD increase in TSHI, the risk of HTG increased by 19.1% (OR=1.191, *P*=0.06648). The risk of HTG in the highest quartile of TSHI was 1.784 times higher than that in the lowest quartile (OR=1.784, *P*=0.0343). TFQI was not associated with the risk of HTG (*P*>0.05) (**Table 6**, **Figure 7**).

**Table 6:** Logistic regression analysis of the correlation between central thyroid hormone sensitivity and HTG.

Exposure	Non-adjusted, OR (95%CI)	*P* value	Adjust Model I, OR (95%CI)	*P* value	Adjust Model II, OR (95%CI)	*P* value
TT4RI	1.019 (1.005, 1.034)	0.00751	1.023 (1.007, 1.038)	0.00334	1.021 (1.006, 1.037)	0.00703
TT4RI per SD	1.324 (1.078, 1.627)	0.00751	1.387 (1.115, 1.726)	0.00334	1.365 (1.089, 1.711)	0.00703
Q1	1(reference)		1(reference)		1(reference)	
Q2	0.894 (0.523, 1.529)	0.68168	0.924 (0.525, 1.625)	0.78298	0.854 (0.480, 1.522)	0.59287
Q3	1.768 (1.087, 2.876)	0.02178	2.126 (1.270, 3.557)	0.0041	1.984 (1.174, 3.354)	0.01054
Q4	1.712 (1.051, 2.788)	0.03081	1.987 (1.181, 3.342)	0.00971	1.895 (1.112, 3.230)	0.01881
TT4RI quartile continuous	1.258 (1.078, 1.468)		1.333 (1.132, 1.571)		1.321 (1.117, 1.564)	
P for trend	0.00365		0.00059		0.00118	
TSHI	1.669 (1.180, 2.360)	0.00377	1.822 (1.269, 2.616)	0.00114	1.721 (1.185, 2.500)	0.00437
TSHI per SD	1.217 (1.025, 1.446)	0.02488	1.236 (1.032, 1.481)	0.02136	1.191 (0.988, 1.436)	0.06648
Q1	1(reference)		1(reference)		1(reference)	
Q2	1.037 (0.611, 1.761)	0.89259	1.164 (0.667, 2.030)	0.59341	1.137 (0.646, 1.999)	0.65652
Q3	1.881 (1.153, 3.067)	0.01136	2.138 (1.275, 3.585)	0.00398	2.004 (1.182, 3.398)	0.00991
Q4	1.640 (0.999, 2.692)	0.05056	1.872 (1.109, 3.160)	0.01895	1.784 (1.044, 3.049)	0.0343
TSHI quartile continuous	1.227 (1.052, 1.431)		1.275 (1.084, 1.499)		1.253 (1.061, 1.479)	
P for trend	0.00927		0.00326		0.00784	
TFQI	1.692 (1.069, 2.678)	0.02488	1.763 (1.088, 2.858)	0.02136	1.596 (0.969, 2.631)	0.06648
TFQI per SD	1.292 (1.086, 1.536)	0.00377	1.350 (1.127, 1.617)	0.00114	1.312 (1.088, 1.581)	0.00437
Q1	1(reference)		1(reference)		1(reference)	
Q2	2.396 (1.430, 4.014)	0.00091	2.943 (1.697, 5.101)	0.00012	2.884 (1.650, 5.038)	0.0002
Q3	1.538 (0.896, 2.641)	0.11867	1.640 (0.928, 2.899)	0.08849	1.586 (0.888, 2.832)	0.11881
Q4	2.132 (1.266, 3.589)	0.0044	2.386 (1.375, 4.141)	0.00199	2.238 (1.272, 3.937)	0.00521
TFQI quartile continuous	1.171 (1.005, 1.366)		1.185 (1.010, 1.392)		1.159 (0.983, 1.367)	
P for trend	0.0433		0.03793		0.07938	

Model 1 adjusted for age, gender, and BMI.

Model 2 Adjusted for age, gender, BMI, FBG, ALT, AST, GGT, TBIL, SUA and SCr. OR, odd ratios; CI, confidence interval; *P* for trend was calculated using the quartiles as continuous variables.

## Discussion

Our study provided evidence of the association between decreased central TH sensitivity and increased TG, and may increase the risk of HTG in euthyroid population. TFQI was positively correlated with TG in males, while TT4RI and TFQI were positively correlated with TG in females; TT4RI, TSHI and TFQI were positively correlated with TG in population with age<65 years and BMI<28 kg/m^2^. This suggested that this relationship between central TH sensitivity and TG was more pronounced in young and middle-aged non-obese adults. The indexes of central TH sensitivity may provide more information than the absolute values of TSH, FT3 and FT4, and our study provided new evidence about THs as independent risk factors for HTG in general population. ​​However, as a fundamental constraint of the cross-sectional design, our findings cannot establish causal relationship—whether impaired TH contributes to HTG development.

Elevated levels of TG are the results of overproduction and impaired clearance of triglyceride-rich lipoproteins-very low-density lipoproteins (VLDL) and chylomicrons (20). HTG is a significant contributor to atherosclerosis and the risk of pancreatitis. Experimental findings suggested that HTG accompanied by increased serum fatty acid (FA) concentration inhibited the functions of mitochondrial complexes in pancreatic alveolar cells (21). This inhibition led to pathologically elevated intracellular calcium, cytokine release, tissue damage and reduced pancreatic ductal function, ultimately resulted in acute pancreatitis (22). HTG was associated with elevated triglyceride-rich lipoprotein (TGRL) and its residues (23), which penetrated the endothelium and interacted with macrophages, leading to the formations of foam cells in the arterial wall and the inflammatory response which would promote atherogenesis (24). In conclusion, patients with HTG may experience various endocrine and metabolic disorders, deserving more in-depth researches.

THs play important roles in regulating lipid metabolism. Evidences suggested that THs, in addition to stimulating lipid substrate utilization by increasing the mobilization of stored TG in adipose tissue, were major regulators of lipid metabolism (25). They promoted lipid mobilization, degradation and *de novo* FA synthesis in the liver (26). THs stimulated 3-hydroxy-3-methylglutaryl-coa reductase (27), which initiated TC biosynthesis and regulated TC metabolism by increasing the expression of sterol regulatory element-binding protein-2 (SREBP-2) (28). Previous studies have shown that the concentration of THs was correlated with hepatic TG content by increasing cholesteryl ester transfer activity, which facilitated the exchange of cholesteryl esters between very low-density lipoproteins (VLDL) and TG in opposite direction (29). Triiodothyronine (T3) could stimulate lipoprotein lipase, which catabolized TG-rich lipoproteins, ultimately leading to a reduction in TG levels (30). In a mouse model with TSH receptor, we could see that TSH inhibited the expression of adipose triglyceride lipase (ATGL) significantly in mature adipocytes in a dose-dependent manner via the PKA pathway (31). However, all of these mechanisms were obtained through animal experiments, which may not be able to fully explain the phenomena observed in our studied population.

Previous studies have confirmed the adverse effects of impaired sensitivity to central THs, with increased incidence of MAFLD (32) and increased prevalence of hyperuricemia (33), as well as dyslipidemia (34). Our findings provided a possible biological basis for the clinical phenomenon where patients with dyslipidemia often exhibited subtle perturbations in thyroid axis, but no overt dysfunction criteria. The stronger associations observed in non-obese and younger adults aligned with population-based evidence that TH sensitivity progressively declined with aging and adiposity (35). Notably, the inflection points (TT4RI=25.8; TFQI=-0.194) in our study may reflect possible clinical cutoffs of evaluating TH-lipid relationships (12).​ Current guidelines (20) primarily focus on LDL-C reduction, while residual HTG-related cardiovascular risk persists in 15-20% in statin-treated patients (4). Our study imply that TH​​ resistance indices may supplement existing tools by unmasking occult endocrine contributors to TG dysregulation, particularly in patients with recurrent pancreatitis or premature ASCVD despite with adequate LDL control.​​ Our findings provided a possible mechanism for the ​​characteristic TH profile​​ observed in patients with dyslipidemia in general population.

Considering the well-established alterations in thyroid profiles with aging (35) and the impact of sex steroids on thyroid function modulation (36), subgroup analyses were conducted based on sex and age. Regarding the differences within gender subgroups, which may be attributed to the different regulatory effects of gonadal hormones on thyroid function. As an example, estrogen or androgen therapy had contrasting impact on the concentration of serum thyroxine-binding globulin, thereby exerting different influence on thyroid function (37). Lipid abnormalities may be rapidly elevated with biological aging and endocrine changes associated with menopause.

Our findings also reveal distinct relationships between central TH sensitivity and TG in distinct BMI statuses. It has been shown that obese individuals exhibited greater central resistance to thyroid hormones, which may be related to the decreased expression of THs and thyrotropin receptors in adipocytes (38). Additionally, interactions between THs and leptin maybe as follows: Leptin secreted by adipose tissue directly stimulates thyrotropin-releasing hormone (TRH) transcription in the hypothalamic paraventricular nucleus (39), while also indirectly promotes TRH secretion via proopiomelanocortin (POMC) products in the arcuate nucleus (40). Despite these potential mechanisms, the precise links between central TH sensitivity and TG across different subgroups remain unexplored, necessitating future investigations.

Certain limitations were presented in our study: 1. Due to the limitation of a cross-sectional study, the causal relationship between central thyroid hormone sensitivity and TG could not be established. 2. This was a single-center study with a relatively small sample size and geographic limitation, and the generalizability of the results still need to be verified in large-scale population. 3. The histories taking in our study may be insufficient and medication histories may not be detailed. 4. We measured the levels of thyroid hormones, but did not test for thyroid hormone antibodies, so we could not completely exclude the effects of Hashimoto’s thyroiditis and other thyroid diseases. 5. We lacked dietary and physical activity data, which were known to influence TG levels and may confound the observed associations.

## Conclusion

Impaired central TH sensitivity was associated with HTG, this association was stronger in young and middle-aged non-obese individuals. We speculated that impaired central TH sensitivity may be contribute to an increased risk of HTG, which still need to be confirmed by more in-depth studies.

## Data Availability

The original contributions presented in the study are included in the article/**Supplementary Material**. Further inquiries can be directed to the corresponding author.
